# The Pharmacological or Genetic Blockade of Endogenous *De Novo* Fatty Acid Synthesis Does Not Increase the Uptake of Exogenous Lipids in Ovarian Cancer Cells

**DOI:** 10.3389/fonc.2021.610885

**Published:** 2021-04-13

**Authors:** Thomas W. Grunt, Lisa Lemberger, Ramón Colomer, María Luz López−Rodríguez, Renate Wagner

**Affiliations:** ^1^ Cell Signaling and Metabolism Networks Program, Division of Oncology, Department of Medicine I, Medical University of Vienna, Vienna, Austria; ^2^ Comprehensive Cancer Center, Vienna, Austria; ^3^ Ludwig Boltzmann Institute for Hematology and Oncology, Vienna, Austria; ^4^ Clinical Research Program, Department of Medical Oncology, Hospital Universitario La Princesa and Spanish National Cancer Research Centre (CNIO), Madrid, Spain; ^5^ Departamento de Química Orgánica I, Facultad de Ciencias Químicas, Universidad Complutense de Madrid, Madrid, Spain

**Keywords:** CD36, FASN inhibitors, fatty acid synthase (FASN), fatty acid uptake, LDL, lipid uptake, ovarian cancer, resistance

## Abstract

Ovarian cancer(OC) is a serious threat to women worldwide. Peritoneal dissemination, ascites and omental metastasis are typical features for disease progression, which occurs in a micro-environment that is rich in high-energy lipids. OC cells require high amounts of lipids for survival and growth. Not only do they import lipids from the host, they also produce lipids *de novo*. Inhibitors of fatty acid(FA) synthase(FASN) – the rate-limiting enzyme of endogenous FA synthesis that is overexpressed in OC – induce growth-arrest and apoptosis, rendering them promising candidates for cancer drug development. However, cancer researchers have long hypothesized that the lipid deficiency caused by FASN inhibition can be circumvented by increasing the uptake of exogenous lipids from the host, which would confer resistance to FASN inhibitors. In contrast to a very recent report in colorectal cancer, we demonstrate in OC cells (A2780, OVCAR3, SKOV3) that neither FASN inhibitors (G28UCM, Fasnall) nor FASN-specific siRNAs can stimulate a relief pathway leading to enhanced uptake of extrinsic FAs or low density lipoproteins (LDLs). Instead, we observed that the growth-arrest due to FASN inhibition or FASN knock-down was associated with significant dose- and time-dependent reduction in the uptake of fluorescently labeled FAs and LDLs. Western blotting showed that the expression of the FA receptor CD36, the LDL receptor(LDLR) and the lipid transport proteins fatty acid binding proteins 1–9 (FABP1–9) was not affected by the treatment. Next, we compared experimental blockade of endogenous lipid production with physiologic depletion of exogenous lipids. Lipid-free media, similar to FASN inhibitors, caused growth-arrest. Although lipid-depleted cells have diminished amounts of CD36, LDLR and FABPs, they can still activate a restorative pathway that causes enhanced import of fluorophore-labeled FAs and LDLs. Overall, our data show that OC cells are strictly lipid-depend and exquisitely sensitive to FASN inhibitors, providing a strong rationale for developing anti-FASN strategies for clinical use against OC.

## Introduction

With more than 140,200 deaths every year, ovarian cancer (OC) represents the most lethal malignancy of the female reproductive tract worldwide (1, 2). Recent progress in anticancer drug development has improved the five-year survival of OC, but the overall mortality yet remains at a stagnant high (3). Thus, novel approaches for treatment of OC are urgently needed. Advanced-stage OC usually spreads to the abdominal cavity and results in floating cells that condition the peritoneal epithelium to produce large volumes of ascites. This fluid typically contains high-energy nutrients like FA and lipids, which can support the growth of OC cells (4). Remarkably, it has been shown that exfoliated OC cells can cross-talk directly with the host’s stromal fibroblasts and omental adipocytes to obtain host lipids from the omental fat pad and to condition the microenvironment for subsequent nidation into the omental tissue – a preferred site for metastasis of OC, which is a major clinical complication (5–7). In addition, OC cells are known to express high levels of fatty acid synthase (FASN), the rate-limiting enzyme for endogenous *de novo* production of FAs (8). The growth and metastasis of OC cells therefore requires adequate supply with intrinsic and extrinsic FA and lipids, which is a potential Achilles’ heel to be targeted with novel anticancer drugs (9). Accordingly, we and others have shown that FASN antagonists can reduce the growth and survival of OC cells (2, 10). Cancer cells exposed to FASN blockers can, however, activate regulatory bypass loops that compensate for the ceased endogenous lipid supply. Thus, while this has not been shown directly until recently, the efficacy of FASN inhibitory drugs could be compromised by activating pathways that stimulate the uptake of extracellular FAs and lipids (11, 12). Accordingly, a very recent report demonstrated that inhibition of FASN upregulated the lipid receptor CD36 and stimulated the uptake of a lipophilic fluorochrome in colorectal cancer cells (13). In contrast, here we show for the first time that this appears not to be the case in OC. We observed that FASN inhibitors do neither affect the expression of lipid uptake- and transport-proteins nor enhance the uptake of exogenous FAs and lipids after short-term exposure. Instead, when given continuously over one or two days, they rather diminish FA- and lipid-import. Therefore, it appears that stimulation of dietary lipid uptake is not an important mechanism for resistance of OC to FASN inhibitors. These finding will be important for future clinical trials with anti-FASN drugs.

## Materials and Methods

### Cells, Culture Conditions and Reagents

OC cell lines A2780 (M. Krainer, Medical University Vienna, Austria), OVCAR3 and SKOV3 (ATCC, Manassas, VA) were maintained in RPMI1640 or α-MEM (Gibco, Karlsruhe, Germany) (14). Media were supplemented with 10% fetal calf serum (FCS) containing 100 IU(μg)/ml penicillin–streptomycin and 2 mM glutamine (Gibco). Cultures were maintained at 37°C, 5% CO_2_ and 95% humidity and cells were tested for absence of viral/bacterial/fungal/mycoplasmal infection (Venor GeM, Minerva Biolabs, Berlin, Germany). The species origin was proven by species-PCR, and cell line identity was examined by fluorescent nonaplex-PCR of short tandem repeat markers (DSMZ, Braunschweig, Germany). FASN inhibitors G28UCM (R. Colomer, M.L. López Rodríguez, Madrid, Spain) (15–17) and Fasnall (T.A.J. Haystead, Durham, NC; J.J. Kwiek, Columbus, OH) (18) were dissolved in pure DMSO and stock solutions were diluted 1:1000 in media.

### RNA Interference

A2780 cells were transiently transfected with 30 or 60 nM FASN-siRNAs according to the manufacturer’s instructions (Dharmacon, Lafayette, CO). Briefly, 3 × 10^5^ cells/35-mm dish were incubated in DharmaFECT-3 siRNA lipid transfection reagent either containing corresponding concentrations of siGENOME nontargeting siRNA #2 (D-001210-02) or containing a set of 4 siRNA species, each at a concentration of 7.5 or 15 nM, that target the mRNA of FASN (ON-TARGETplus SMARTpool FASN, L-003954-00). Unselected polyclonal transfectant populations were cultured for 72 h at 37°C, 5% CO2, and 95% humidity in RPMI-1640 containing 5% FCS and 2 mM glutamine and then lysed and processed for SDS-PAGE and Western blotting to demonstrate knock-down of FASN target protein expression or subjected to a cell proliferation assay as described below.

### Western Blotting

**Figure 1:**
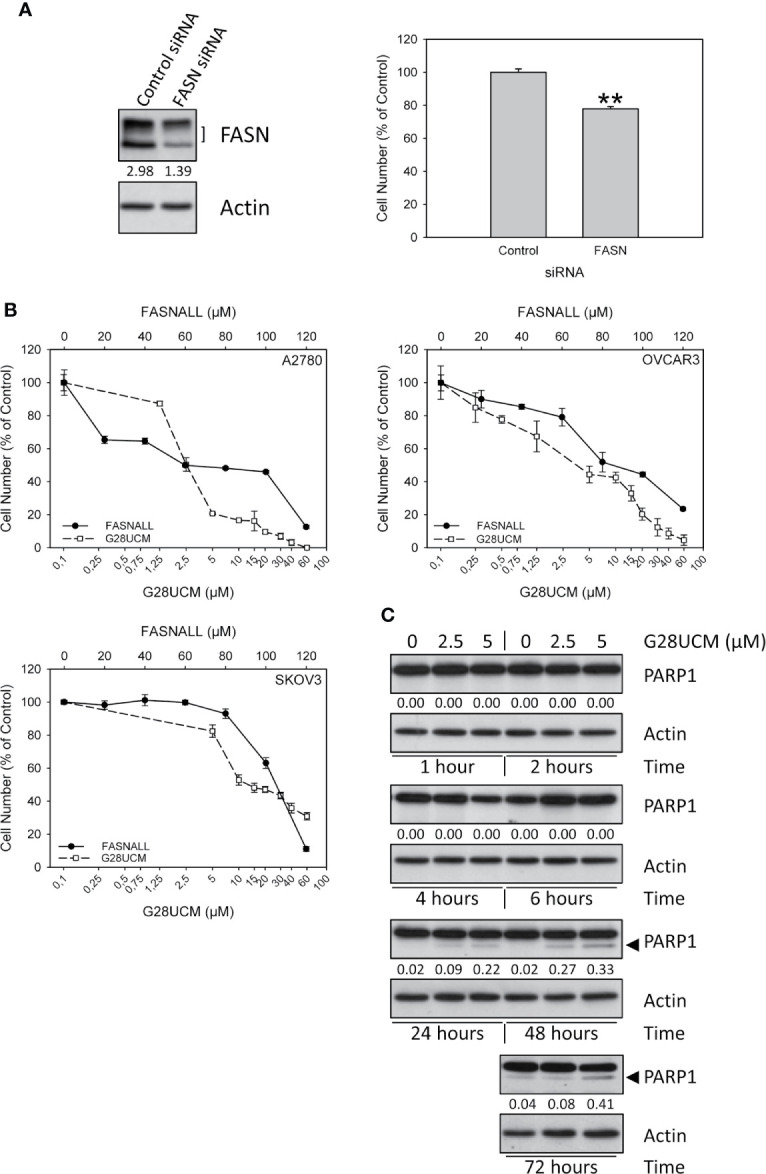
Growth of OC cells is dependent on FASN. **(A)** Down-regulation of FASN protein expression by transfection of FASN siRNAs (30nM) was proven by Western blotting (left, one of three independent experiments) and was found to correlate with reduction of cell growth in A2780 cells (right). Results were obtained after 72 hours of incubation and are expressed in % of Control. Means ± SD, n = 3. One-tailed Student’s t-test at p < 0.01 (**) between cells transfected with non-targeting control siRNA (Control) or with FASN-targeting siRNA (FASN). **(B)** The FASN inhibitors G28UCM and Fasnall dose-dependently inhibit *in vitro* growth of A2780, OVCAR3 and SKOV3 OC cell lines after 72 hours of drug exposure. Note that concentrations of G28UCM are on a logarithmic, while Fasnall concentrations are on a linear scale. Formazan dye assay, means ± SD, n = 3. **(C)** Western blot analysis of dose- and time-dependent induction of apoptosis by G28UCM in A2780 OC cells as demonstrated by expression of PARP1 cleavage products (◄). Actin was used as loading control. The band intensities were determined using ImageJ software and the ratios between FASN (both bands in the left panel in **(A)** marked with]) or PARP1 cleavage products (◄ in **C**) and actin were given under the FASN **(A)** or PARP1 **(C)** autoradiographs. One of two independent determinations is shown.

**Figure 2:**
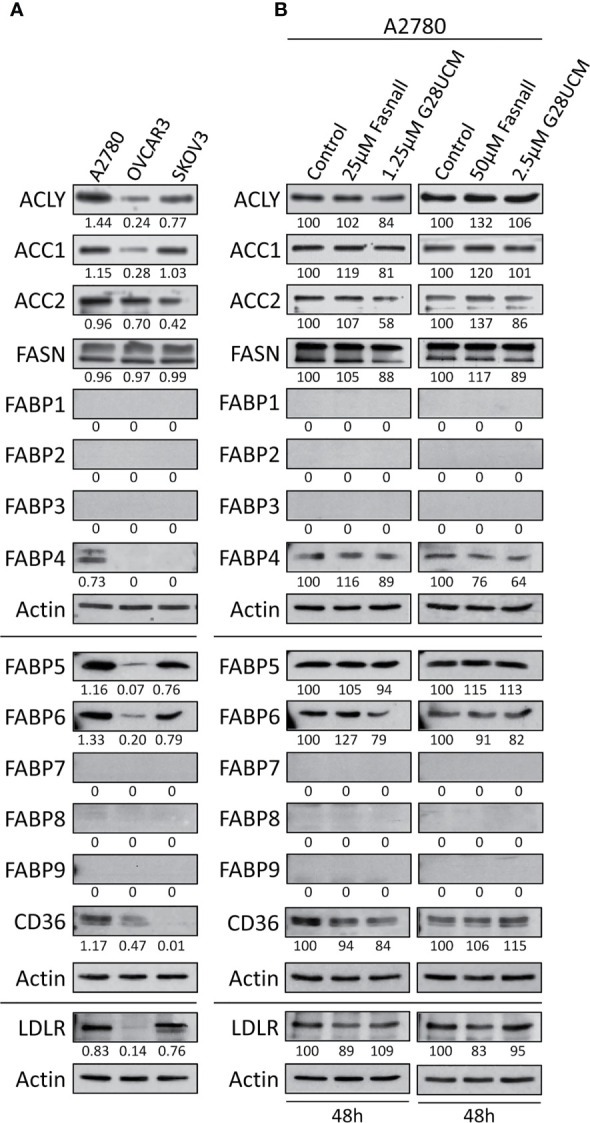
Expression of lipid metabolic proteins in OC cell lines as determined by Western blotting. **(A)** Baseline levels of the lipogenic enzymes ATP-citrate lyase (ACLY), acetyl-CoA carboxylase 1 (ACC1), ACC2 and FASN, the lipid transport proteins FABP1 – FABP9, and the lipid receptor proteins CD36 and LDLR in A2780, OVCAR3 and SKOV3 OC cell lines. A2780 cells were found to express the widest range of these lipid metabolic proteins. **(B)** Inhibition of FA synthesis by the FASN inhibitors G28UCM or Fasnall does not affect the expression of the lipid metabolic proteins in A2780 OC cells. Blots were (co-)probed for several proteins of interest and re-probed for actin as loading control. Previous antibodies were stripped from the membranes before detection of ACLY, ACC1 and ACC2. The band intensities were determined using ImageJ software, and in A the ratios between the proteins of interest and the corresponding actin are given under each autoradiograph, whereas in B these ratios were normalized to the untreated control samples (100%) and are expressed in percent of control. Experiments were repeated at least once.

Untransfected or siRNA transfected OC cells were allowed to attach overnight and were grown for 48 or 72 hours in media containing 5% FCS ± FASN inhibitor (G28UCM or Fasnall) or 5% FCS that has been depleted of lipids (Biowest, Nuaillé, France). After lysis, proteins were subjected to SDS–PAGE, blotted and immunostained as described (19, 20) using anti-FASN (BD Biosciences, San Jose, CA; 1:500), anti-ATP-citrate lyase (ACLY), anti-acetyl-CoA carboxylase 1 (ACC1), anti-ACC2, anti-PARP1, anti-FABP1, anti-FABP3, anti-FABP4, anti-FABP5, anti-FABP7, anti-CD36 (Cell Signaling Technology, Boston, MA; 1: 200–1:3 000), anti-FABP2, anti-FABP6, anti-FABP9, anti-LDLR (Abcam, Cambridge, UK; 1: 200–1: 1 000), anti-FABP8 (Thermo Fisher, Waltham, MA; 1: 200), and anti-actin (Santa Cruz Biotechnology, Dallas, TX; 1:1 000). Secondary antibodies were peroxidase-labeled donkey-anti-rabbit (Abcam), or donkey anti-goat IgG (Santa Cruz Biotechnology) at 1: 15 000, or donkey-anti-mouse (Jackson ImmunoResearch, West Grove, PA), or donkey anti-sheep (Thermo Fisher) at 1:10 000. Detection was performed with enhanced chemiluminescence. In some cases, polyvinylidene difluoride membranes had to be stripped from previous antibodies using a mild procedure (2 x 10 min incubation of the membrane in 1.5% glycine, 0.1% SDS, 1% Tween20, pH 2.2 at room temperature, followed by 2 washes in PBS and 2 in TBST) before blocking and re-probing. The band intensities were determined by ImageJ software (NIH, Bethesda, MD) and changes in the level of the proteins of interest were normalized to actin as loading control detected on the same membranes. Obtained ratios are directly given (**Figures 1** and **2A**) or are expressed in % of untreated control (**Figure 2B**), or relative to cultures grown for 48 hours in 5% FCS + lipids (**Figure 6C**). Corresponding full membrane images are given in the **Supplementary Material** section.

### Cell Proliferation

Untransfected or siRNA transfected OC cells (1,500–7,500/well, 96-well plate) were cultured overnight in medium containing 5% FCS to let them adhere, before media containing 5% FCS ± various concentrations of FASN inhibitor (G28UCM or Fasnall) or 5% lipid depleted FCS was added. After the indicated periods of time, cell numbers were either determined with a formazan dye assay (Biomedica, Vienna, Austria) as described (21–23) or were directly counted in a hemocytometer under the microscope.

#### Cell Viability and Apoptosis

Trypsinized cells were stained with trypan blue (0.4% in PBS) and the cell viability was determined by dividing the number of unstained (viable) cells by the sum of unstained and stained (dead) cells, and was expressed in % of total cells.

Drug-induced apoptosis was determined by Western blotting to detect the appearance of cleaved PARP1 fragments – a hallmark of programmed cell death – according to previously published protocols (2).

### FA and LDL Uptake Assays

Wildtype or siRNA transfected OC cells (7,500/well; black 96-well plate with optical bottom, Nunc, Roskilde, Denmark) were allowed to adhere overnight. Non-transfected cells were then exposed for 48 hours to increasing concentrations of G28UCM or Fasnall in media containing 5% FCS and dose-dependent changes in FA uptake rates were determined. Concentrations that caused at least 50 – 60% alteration of FA and LDL uptake were applied for subsequent time-course analyses after 1, 2, 4, 24 and 48 hours of drug exposure. At the end of drug treatment or after 72 hours of exposure to siRNAs, cells were extensively washed and further processed for fluorescent labeling. To evaluate the effect of lipid starving on FA and LDL uptake, A2780 cells were allowed to adhere overnight in culture media containing 5% FCS. Then, the cells were extensively washed and cultured for 1, 2, 4, 24 and 48 hours in media supplemented with 5% lipid-depleted FCS before the BODIPY-dodecanoic acid fluorescent fatty acid analog (BODIPY 500/512 C1, C12; contained in the QBT™ Fatty Acid Uptake Kit from Molecular Devices, San Jose, CA) or the pH-sensitive fluorochrome pHrodo™ Red conjugated to LDL (pHrodo™ Red LDL Uptake Kit from Thermo Fisher) was added in media containing 0%, 1% or 5% FCS. After incubation for about 10 min (FA uptake) or 4 hours (LDL uptake) the fluorescence signals were determined according to the manufacturers protocols in a SpectraMax iD3 multi-mode microplate reader (Molecular Devices). Obtained fluorescence values were normalized to cell number, determined by formazan dye assay and hemocytometer counting that were performed in parallel. Vehicle(0.1% DMSO)-treated control samples grown in medium supplemented with 5% FCS were set to 100% and experimental results were expressed in percent of control.

### Statistical Analysis

Data are presented as mean values ± standard deviation of triplicate experiments. Where appropriate, statistically significant differences between control and experimental groups were determined by one-tailed Student’s t-test or by using ANOVA followed by a *post hoc* Scheffe test at levels of significance of p < 0.05 (*), p < 0.01 (**) and p < 0.001 (***).

## Results

### The Growth of OC Cells is Dependent on the Expression and Activity of FASN

In accordance with previous reports (2, 21, 22) we show that disabling FASN reduces OC cell growth. **Figure 1A** demonstrates that even a moderate down-regulation of FASN transcript expression by FASN-specific small interfering RNA significantly decreases the growth of A2780 OC cells. Full images of Western blot autoradiographs showing residual FASN protein expression after siRNA-mediated partial knock-down are given in **Supplementary Figure S1**. Dependence of cell growth on FASN activity was further corroborated by pharmacologic blockade in three different OC cell lines using the FASN-specific inhibitors G28UCM and Fasnall. Both drugs caused rapid and dose-dependent inhibition of proliferation in A2780, OVCAR3 and SKOV3 OC cells as demonstrated by formazan dye assay (**Figure 1B**) and direct microscopic cell counting (**Supplementary Figures S2A, B**). In agreement with previous results (2), Western blot analysis demonstrated the appearance of cleaved PARP1 fragments after 24 hours of drug exposure, a hallmark of programmed cell death, corroborating induction of apoptosis (**Figure 1C**), while trypan blue dye exclusion did not reveal unspecific drug toxicity (**Supplementary Figures S2C, D**). G28UCM is a synthetic derivative of epigallocatechin gallate (EGCG). It interacts with the β-ketoacyl reductase (KR) domain of FASN (24–26). Fasnall, on the other hand, is a thiophenopyrimidine that has been designed to specifically interfere with NADPH co-factor binding (18). Because of these distinct mechanisms of action, the IC_50_ values of the two drugs were found to be significantly different to each other (**Table 1**). Note that in **Figure 1B**, the concentrations of G28UCM are on a logarithmic scale while the Fasnall concentrations are linear. The slopes of the dose-response relationships are apparently quite different to each other indicating distinct widths of the therapeutic windows. Nevertheless, both drugs have been found to be well tolerated *in vivo* (18, 26). Moreover, imidazopyridine-based compounds such as TVB-2640 are currently in clinical studies. One of its derivatives, TVB-3664, reveals significant anti-proliferative activity in OC cells as shown in **Supplementary Figure S2E**.

**Table 1:** IC_50_ values and their quotient (ratio of equivalence) as metrics for growth inhibition by FASN inhibitors Fasnall and G28UCM in A2780, OVCAR3, and SKOV3 OC cells as determined with a formazan dye assay.

	A2780	OVCAR3	SKOV3
	**IC_50_ (µM)** (Means ± SD)		
**Fasnall**	55.1 ± 0.4	88.6 ± 3.4	105.0 ± 1.0
**G28UCM**	2.6 ± 1.7	4.5 ± 0.6	10.7 ± 5.8
	**Ratio of Equivalence (IC_50 Fasnall_/IC_50 G28UCM_)**		
**Fasnall/G28UCM**	21.2	19.7	9.8

Means ± SD of at least three independent experiments (for details see Materials and Methods section).

### Blockade of *De Novo* Fatty Acid Synthesis Has No Effect on the Expression of Lipid Metabolic Proteins in OC Cells

Binding and import of FAs at the plasma membrane and their intracellular transport are complex processes and involve numerous multifunctional proteins including CD36 (aka fatty acid translocase, FAT), which acts as a receptor for a wide range of ligands such as phospholipids and long-chain FAs. Low-density lipoprotein (LDL), the major cholesterol-carrying lipoprotein, on the other hand, will be taken up by endocytosis *via* the LDL receptor (LDLR). Subsequent intracellular trafficking of FAs is managed in human cells by members of a family of nine distinct FA-binding proteins (FABP1 – FABP9), which are expressed in a tissue-specific pattern (27).

Western blot experiments showed that A2780 cells contain the widest range of lipid metabolic proteins including high level-expression of ACLY, ACC1, ACC2, FASN, FABP4, FABP5, FABP6, CD36, and LDLR. In contrast, OVCAR3 cells contain only very low amounts of ACLY, ACC1, FABP5, FABP6, CD36, and LDLR, while SKOV3 showed intermediate content of ACLY, ACC1, ACC2, FABP5, FABP6, and LDLR. None of the other FABPs (FABP1, FABP2, FABP3, FABP7, FABP8, FABP9) were identified (**Figure 2A**). A2780 cells thus contain the richest repertoire of lipid metabolic enzymes. We wondered whether inhibition of *de novo* fatty acid synthesis would change the expression of these enzymes. Western blots shown in **Figure 2B**, demonstrate that neither G28UCM nor Fasnall could alter the levels of ACLY, ACC1, ACC2, FASN, FABP1 – FABP9, CD36, and LDLR in A2780 cells. This suggests that blockade of lipogenesis does not modulate the steady state levels of crucial lipid metabolic proteins. Full images of the Western blot autoradiographs are shown in **Supplementary Figures S3A–C**.

### Blockade of *De Novo* FA Synthesis Does not Stimulate Uptake of Exogenous Lipids into OC Cells

**Figure 3:**
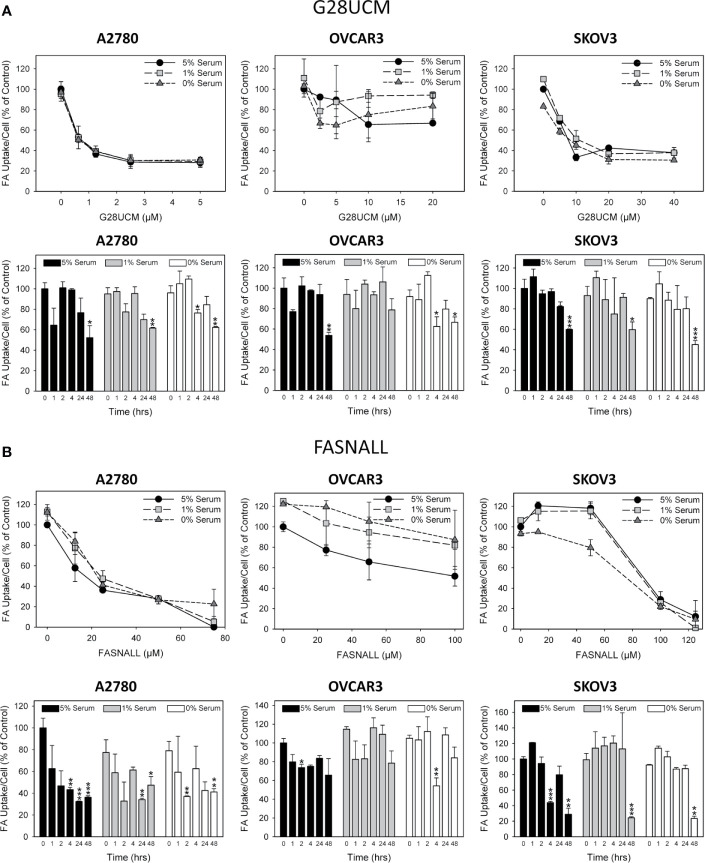
The effect of inhibition of endogenous *de novo* fatty acid synthesis on the uptake of exogenously supplied FAs in OC cells. A2780, OVCAR3 and SKOV3 cells were exposed for 48 hours to increasing concentrations of the FASN inhibitors G28UCM **(A)** or Fasnall **(B)** before FA uptake was determined by the QBT™ Fatty Acid Uptake Kit as described in Materials and methods. Obtained dose-response curves (upper panels in **A**, **B**) were used to determine optimal drug concentrations (G28UCM: 1.25, 2.5, 10 µM for A2780, OVCAR3 and SKOV3; Fasnall: 25, 50, 100 µM for A2780, OVCAR3 and SKOV3) for subsequent time courses (lower panels in **A**, **B**). Comparison of the data in the presence of 5%, 1% or 0% serum reveals that withdrawal of concurrent lipid contained in the serum could not reverse the drug-mediated reduction of uptake of the fluorescent dodecanoic FA analog BODIPY 500/512 C1, C12. Fluorescence signals were normalized to cell numbers that have been determined in parallel by formazan dye cell proliferation assays and were expressed in % of vehicle(0.1% DMSO)-treated control cells grown in the presence of 5% serum. Means ± SD, n = 3. ANOVA followed by Scheffe test, p < 0.05 (*), p < 0.01 (**) and p < 0.001 (***) relative to vehicle-treated cells grown in the presence of the corresponding concentration (5%, 1% or 0%) of serum.

**Figure 4:**
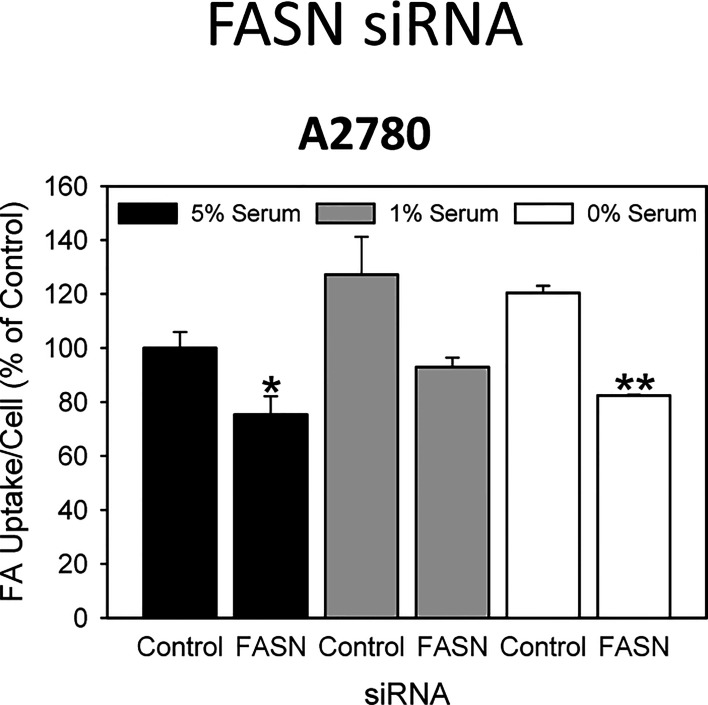
A 48-hours knock-down of FASN by specific siRNAs inhibits the uptake of exogenously supplied FA. Comparison of the data in the presence of 5%, 1% or 0% serum reveals that reduction of concurrent lipid contained in the serum can support the uptake of the fluorescent dodecanoic FA analog BODIPY 500/512 C1, C12 in the untreated control cells, but cannot reverse drug-mediated reduction of FA uptake. Fluorescence signals were normalized to cell numbers that have been determined in parallel by formazan dye cell proliferation assays and were expressed in % of vehicle(0.1% DMSO)-treated control cells grown in the presence of 5% serum. Means ± SD, n = 3. One-tailed Student’s t-test at p < 0.05 (*) and p < 0.01 (**) between cells transfected with FASN-targeting siRNA (FASN) or with non-targeting control siRNA (Control) grown in the presence of the corresponding concentration (5%, 1% or 0%) of serum.

It is widely believed that cancer cells attempt to compensate for the lipid deficiency caused by inhibition of *de novo* fatty acid synthesis by stimulating uptake of lipids from the microenvironment. However, the data presented here does not support this view. As demonstrated in **Figure 3**, both G28UCM and Fasnall cause a dose- and time-dependent reduction in the uptake of fluorescently labeled FA in all 3 OC cell lines tested, with A2780 being the most and OVCAR3 being the least sensitive to the FASN inhibitors (**Figures 3A, B**). Time-course experiments revealed that the effect was slowly mounting. It required at least 24 hours of drug exposure and was not preceded by a transient increase in FA uptake. Moreover, knock-down of FASN with siRNA also significantly reduced FA uptake in A2780 cells (**Figure 4**). Notably, the decrease in fluorescent FA uptake was very robust and could not be reversed by removing natural lipids concurrently present in the serum (**Figures 3** and **4**).

**Figure 5:**
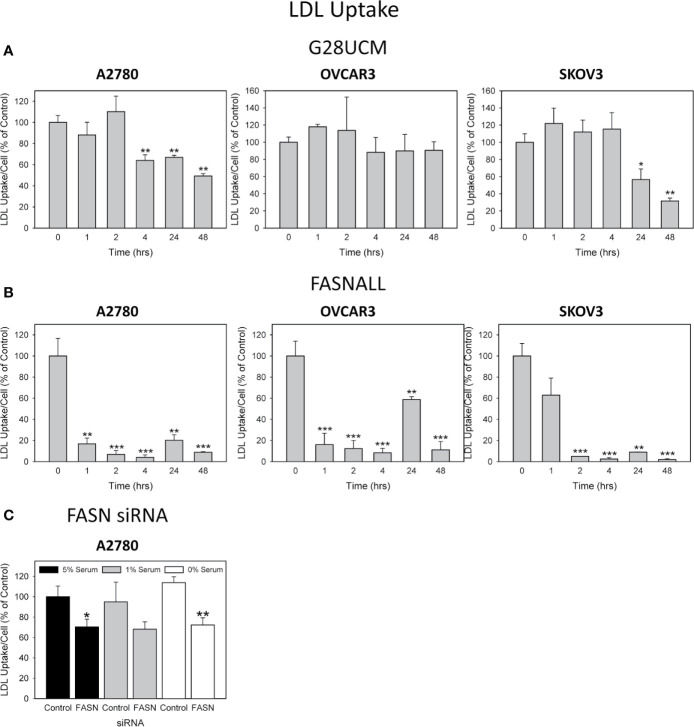
The effect of inhibition of endogenous de novo fatty acid synthesis on the uptake of exogenously supplied LDLs in OC cells. A2780, OVCAR3 and SKOV3 cells were cultured in media containing 1% serum and were exposed for 0 – 48 hours to the optimal concentrations (as determined in **Figure 3**) of the FASN inhibitors G28UCM (1.25, 2.5, 10 µM for A2780, OVCAR3 and SKOV3) **(A)** or Fasnall (25, 50, 100 µM for A2780, OVCAR3 and SKOV3) **(B)** before LDL uptake was determined by the pHrodoTM Red LDL Uptake Kit as described in Materials and methods. Fluorescence signals were normalized to cell numbers that have been determined in parallel by formazan dye cell proliferation assays and were expressed in % of vehicle(0.1% DMSO)-treated control cells. Means ± SD, n = 3. ANOVA followed by Scheffe test, p < 0.05 (*), p < 0.01 (**) and p < 0.001 (***) relative to vehicle-treated cells. **(C)** A 48-hours knock-down of FASN by specific siRNAs inhibits the uptake of exogenously supplied LDL. Comparison of the data in the presence of 5%, 1% or 0% serum reveals that reduction of concurrent lipid contained in the serum can support the uptake of pHrodoTM Red LDL in the control transfectants, but cannot reverse reduction of LDL uptake when FASN was knocked-down. Fluorescence signals were normalized to cell numbers that have been determined in parallel by formazan dye cell proliferation assays and were expressed in % of Control (cells transfected with non-targeting control siRNA). Means ± SD, n = 3. One-tailed Student’s t-test at p < 0.05 (*) and p < 0.01 (**) between cells transfected with FASN-targeting siRNA (FASN) or with non-targeting control siRNA (Control) grown in the presence of the corresponding concentration (5%, 1% or 0%) of serum.

Cells can obtain additional FA by breaking down more complex lipids like cholesterol, which is an integral part of LDLs. Thus, LDLs are another essential source of FA and lipids. As with FA uptake, import of fluorescently labeled LDLs was downregulated by the drugs in the cell lines in a time-dependent manner, as determined by semiquantitative fluorescence spectroscopy (**Figures 5A, B**). Notably, this drug-dependent decrease in LDL uptake occurred more rapidly and was stronger than that of FA uptake (compare **Figures 3**–**5**). The two drugs did not block lipid import with equal efficiency. Fasnall produced much stronger effects than G28UCM, although both were used in equipotent growth inhibiting concentrations according to their ratio of equivalence (**Table 1**).

### Depletion of Exogenous Lipids Inhibits the Growth of OC Cells

**Figure 6:**
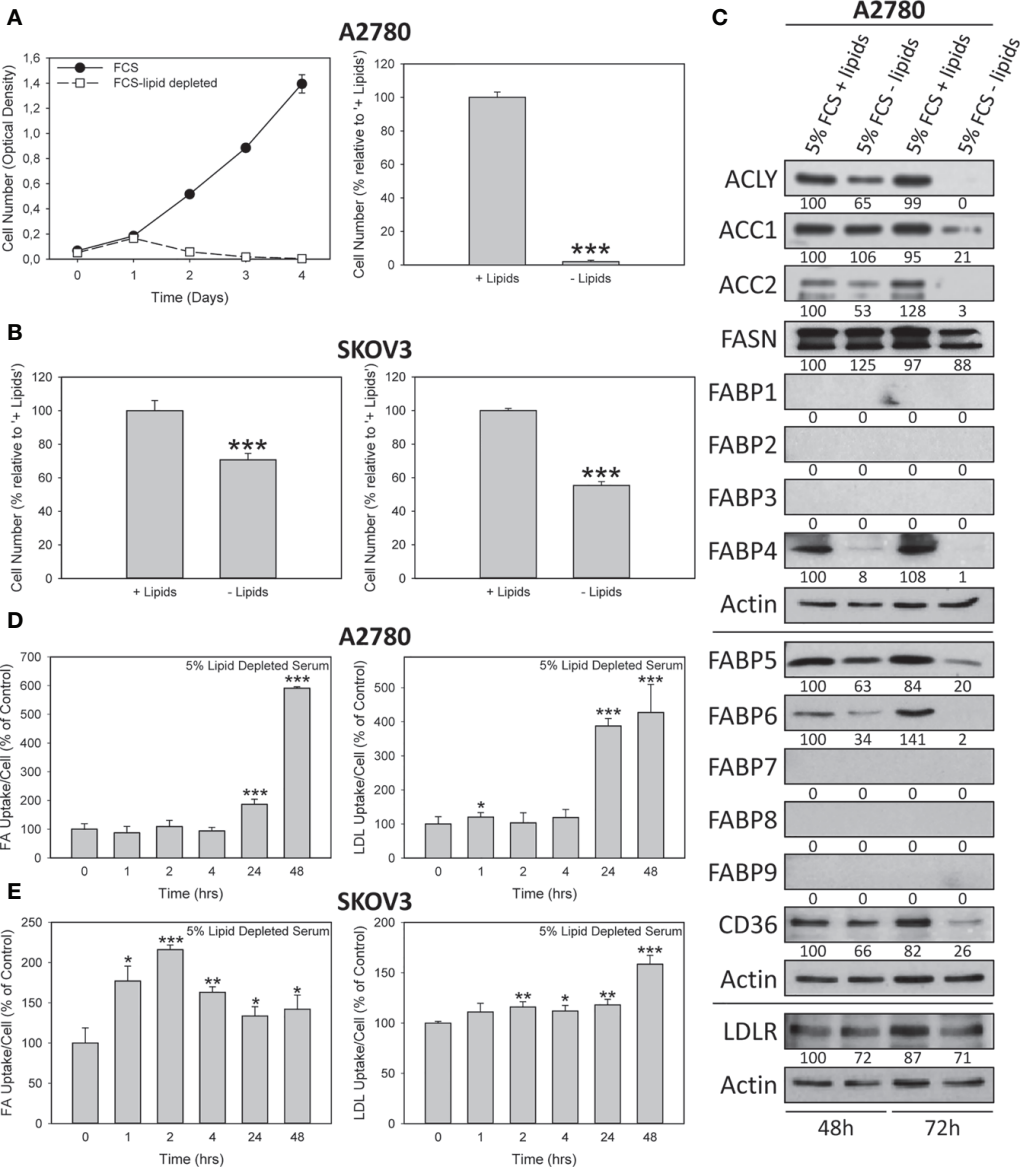
Cultivation of A2780 **(A, C, D)** and SKOV3 **(B, E)** OC cells in lipid-free media inhibits growth **(A, B)**, down-regulates expression of lipid metabolic proteins **(C)** and conditions the cells for restorative import of exogenously supplied FAs or LDLs **(D, E)**. Cells were cultured for 0 – 4 days (A, left panel) or for 72 hours (**A**, right panel; **B** both panels) in media containing 5% FCS with or without lipids. Analysis of cell growth was either by formazan dye assay (**A** both panels; **B** left panel) or direct microscopic counting in a hemocytometer (**B** right panel). Western blots **(C)** were (co-)probed for lipid metabolic proteins, stripped if necessary, and re-probed for actin as loading control. The band intensities obtained after chemoluminescence detection of autoradiographs were determined using ImageJ software and the ratios between the proteins of interest and the corresponding actin on the same membrane were calculated. Horizontal lines separate the different membranes. These ratios were normalized to cultures grown for 48 hours in media supplemented with 5% FCS + lipids (arbitrarily set to 100%) and are given under each autoradiograph in percent. Autoradiographs of one of two experiments are shown. For further technical details of cell proliferation experiments, Western blotting, and uptake assays for FAs and LDLs see Materials and methods. Means ± SD, n = 3. Student’s t-test **(A, B)** or ANOVA followed by Scheffe test **(D, E)**, p < 0.05 (*), p < 0.01 (**) and p < 0.001 (***).

Remarkably, a high level of FASN expression is required but not sufficient for OC cell growth. As shown in **Figures 6A, B**, culturing A2780 or SKOV3 cells in lipid-stripped serum results in a blockade of cell proliferation.

### Depletion of Exogenous Lipids Induce Down-Regulation of Lipid Metabolic Proteins

Removal of exogenous lipids from cell culture serum resulted in a gradual decrease in all lipid metabolic proteins examined in A2780 cells (**Figure 6C**). These proteins are no longer required and are therefore downregulated in an autoregulatory loop that controls the import, transport and remodeling of nutrient lipids in the cells. Full images of the Western blot autoradiographs are shown in **Supplementary Figure S4**.

### Strong Restorative Import of FAs and LDLs After Cultivation Under Lipid-Free Conditions

Lipid deficiencies for periods of up to 24 hours can be well tolerated by the cells before they reduce cell growth (**Figures 6A, B**) and reduce the expression of lipid metabolic proteins (**Figure 6C**). Nevertheless, despite down-regulation of the transport systems, lipid depleted cells can still effectively reactivate their lipid transport when exogenous lipids are supplied. Time course experiments using fluorescence spectroscopy revealed a highly significant increase in the uptake of added fluorescent FAs or LDLs. Interestingly, as shown in **Figures 6D, E**, the dynamics of activation of lipid relief-uptake was significantly different in A2780 and SKOV3 cells. While in A2780 the activation was slow and intense, in SKOV3 it started very quickly, but did not reach such high peak levels. Comparison of the data on growth inhibition shown in **Figures 1B** and **6A, B** may suggest that both conditions – drug-mediated blockade of endogenous FA biosynthesis and depletion of exogenous lipid supply – share the same antiproliferative mechanism. Further analysis, however, revealed crucial differences. In lipid-depleted cells was neither growth arrested in a specific phase of the cell cycle nor was apoptosis induced (**Supplementary Table S1**). Cells exposed to FASN inhibitors, however, experienced both cell cycle arrest and apoptosis (2, 28; **Figure 1C**). Thus, abrogation of lipid uptake after treatment with FASN inhibitors is a consequence rather than a primary mechanism of drug-mediated growth arrest. Interestingly, lipid deficiency was not compensated by upregulating the FA synthesis pathway. This most likely is due to the fact that the baseline activity of this pathway is already maximal in cancer cells (28) and therefore cannot be increased any further. Instead, prolonged lipid depletion results in downregulation of most lipid metabolic enzymes (**Figure 6C**).

## Discussion

Cancer cells show typical aberrations in their cell metabolism. These cancer-specific changes are not limited to glycolytic pathways (aerobic glycolysis or Warburg effect), but also affect other processes. Specific alterations in the lipid metabolism, for example, cause both activation of *de novo* synthesis of endogenous FA and import of exogenous FA. This enables the malignant cells to cope with the high demand for lipids, which serve as building blocks for new membranes, as signaling molecules and as vehicles for bioenergy for growth, migration, invasion and metastasis (4, 29, 30). Disruption of *de novo* FA synthesis therefore represents a promising pharmacological approach to cancer control, and numerous attempts have been made to block the enzymes that regulate FA synthesis, including FASN – the key enzyme in the process.

TVB-2640 is a potent FASN inhibitor that is currently tested in Phase I and II clinical trials against various solid tumors (31). Unfortunately, high *in vitro* sensitivity of cancer cells to FASN inhibitors does not always lead to high *in vivo* sensitivity. While FASN inhibitors are known to be highly effective cancer drugs in cell culture, they are often only moderately effective *in vivo*. Several reasons can contribute to this problem. A prominent hypothesis suggests that tumor cells with impaired FASN function can compensate for lipid deficiency by importing exogenous lipids and FA from the microenvironment. Surprisingly, however, there is still no direct experimental evidence for this intuitive idea other than a single recent study, which showed that colorectal cancer cells exposed to the FASN inhibitor TVB-3664 import large amounts of dietary lipids *via* the FA receptor and transporter protein CD36 to alleviate their lipid shortage (13). TVB-2640, TVB-3664, and G28UCM belong to a group of novel drugs that strongly bind to the β-ketoacyl reductase (KR) domain of FASN and act *via* similar molecular mechanisms. They show equivalent on-target effects and inhibit the *in vitro* and *in vivo* growth of OC and many other solid tumors with IC_50_ concentrations in the µM range (32–35). Interestingly, despite these similarities, treated OC cells, unlike colorectal cancer cells, showed neither CD36 upregulation nor activation of lipid uptake. Accordingly, genetic knock-down of FASN upregulated CD36 and lipid uptake in colorectal cancer, but not in OC cells. A possible reason for this discordance could be that the duration of FASN blockade/interference was significantly different in the two studies. While in colorectal cancer FASN enzyme function was permanently blocked for more than 6 days or the protein was irreversibly knocked-down (13), in OC both enzyme function and protein levels were transiently reduced for a maximum of 72 hours. Extending these periods was not feasible due to treatment-induced cell death in OC. Advanced OC thus appears to be exquisitely dependent on lipid metabolic pathways and to react more sensitively to a blockade of lipid biosynthesis than colorectal cancer. The upregulation of CD36 and lipid uptake represents a long-term compensatory response to the treatment that can be induced in colorectal carcinoma cells but not in OC cells, since in the latter, the cell damage induced by the treatment is too severe to allow activation of relief pathways.

While aerobic glycolysis is downregulated in OC, both *de novo* synthesis of endogenous FA and uptake of exogenous dietary FA are activated in these tumors, which favors malignant growth and dissemination in the peritoneal cavity resulting in ascites formation and abdominal metastasis (2, 22, 28). OC cells have been shown to import exogenous FA from ascites fluid, which serves as a rich source of lipids. In addition, it is known that OC cells settle and preferentially invade omental and splenoportal sites that contain abundant adipose tissue and serve as the main lipid reservoir in the body. Notably, OC cells make direct contact with these adipocytes in order to obtain exogenous lipids and FA from them (7, 36). Targeting production and import of lipids in OC would therefore be a promising strategy both for systemic treatment and for locoregional lavage *via* the peritoneal route. Here we determined for the first time that the anti-cancer effects of a blockade of endogenous *de novo* FA synthesis in OC are not diminished by upregulating exogenous FA- and lipid-uptake. We confirm that the growth of OC cells in *ex vivo* environments is strongly dependent on the expression and activity of FASN. For example, even moderate downregulation of FASN protein after incomplete knock-down of the gene with siRNA led to a significant reduction in cell growth (**Figure 1A**). In addition, both FASN inhibitors, G28UCM and Fasnall, caused potent dose-dependent inhibition of cell growth in all three OC cell lines; albeit the course of the dose-response relationships of the two drugs was quite different. This can be related to their different modes of action. G28UCM interacts with the β-ketoacyl reductase (KR) domain of FASN (24–26), whereas Fasnall disturbs the NADPH co-factor binding (18). Accordingly, their IC_50_ values were significantly different (**Table 1**). Consistent with the fact that OC cells show very active uptake of FA and lipids, we observed that OC cells express a wide repertoire of FA receptor and transport proteins, including CD36, LDLR, FABP4, FABP5 and FABP6 (**Figure 2A**). This confirms that the growth and progression of OC is highly dependent on active lipid metabolism. In the clinical setting, bypass routes that stimulate the compensatory import of extracellular lipids and FA could therefore lower the efficacy of FASN blockers against OC. This can be prevented by blocking both lipid biosynthesis and lipid uptake at the same time. Data presented here indicate that FA uptake and transport pathways are not activated in response to inhibitor-mediated abolition of FA synthesis. For example, the cellular levels of FA binding and transport proteins did not increase in response to FASN inhibitors in A2780 OC cells (**Figure 2B**). Moreover, we observed a significant dose- and time-dependent downregulation of FA uptake in all three tested OC cell lines with both FASN inhibitors, regardless of previous supplementation or depletion of exogenous serum lipids. The downregulation of lipid import was most pronounced in A2780, followed by SKOV3 and OVCAR3 cells (**Figures 3A, B**). Of note, similar results were obtained when FASN was downregulated by siRNA (**Figure 4**). Since LDL is the major source of phospholipids and cholesterol, we strived for detection of LDL import. As shown in this study (**Figure 5**), G28UCM, Fasnall and FASN siRNA inhibited the uptake of LDLs. The effects of Fasnall were particularly pronounced. In order to characterize the dependence of OC cells on lipid metabolism in more detail, we compared the inhibition of endogenous production of lipids with the depletion of exogenous lipids. Withdrawal led to a significant growth reduction, which was accompanied by a loss of FA metabolic proteins (esp. ACLY, ACCs, FABP4 - FABP6, and CD36). This suggests a tight autoregulatory loop for expression of lipid metabolic proteins. Depending on the cell line, lipid-starved cells either compensate for the lack of lipids for several hours before replenishing their pools (A2780) or instantaneously activate the uptake of exogenous lipids (SKOV3). The genetic or pharmacological blockade of FASN exerts cytotoxic effects, which prohibit the activation of FA or LDL relief uptake pathways. Forced lipid starvation, on the other hand, is a physiological condition that can be easily reversed (**Figure 6**). Accordingly, treatment with FASN inhibitors leads to an arrest in the G2/M and/or S phase of the cell cycle and to apoptosis (28 and **Figure 1C**). Lipid deficiency, on the other hand, only causes a reduction of cell proliferation without a specific cell cycle block or induction of apoptosis (**Supplementary Table S1**). This suggests that the decrease in lipid uptake following inhibition of FASN is more a consequence than a cause of FASN inhibitor-induced growth arrest. Similar to our observation, a general slowdown in cell cycle progression in the absence of a complete block has previously been observed in cultures with reduced polyamine levels (37, 38).

Our data show that lipid metabolism is regulated differently in OC than in colorectal carcinoma. In the latter, inhibition of FASN leads to upregulation of CD36 and consequently to an increased uptake of FA (13), whereas CD36 levels remain at baseline (**Figure 2B**) and lipid uptake becomes downregulated in OC. We conclude that OC cells are exquisitely sensitive to interference with FASN function. Thus, the strong effect of FASN blockade in OC prevents the cells from upregulating FA and LDL import and adapting to the stress condition. Instead, the treatment drives the cells into degeneration, which is accompanied by paralysis of the lipid transport. This suggests that combinations of FASN inhibitors with inhibitors of lipid uptake are not necessary in the treatment of OC.

## Data Availability Statement

The original contributions presented in the study are included in the article/**Supplementary Material**. Further inquiries can be directed to the corresponding author.

## Author Contributions

TG conceived and planned the experiments, supervised the project and wrote the paper. LL and RW carried out the experiments. RC and ML-R synthesized FASN inhibitors, performed quality controls and provided G28UCM. TG, LL and RW analyzed the samples and the data. All authors contributed to the article and approved the submitted version.

## Funding

This work was financially supported by the Medical Scientific Fund of the Mayor of the City of Vienna, by the ‘Initiative Krebsforschung’ of the Medical University of Vienna, and by the Herzfelder Familienstiftung, Vienna, Austria.

## Conflict of Interest

The authors declare that the research was conducted in the absence of any commercial or financial relationships that could be construed as a potential conflict of interest.
