# Supplementary material for: The Pharmacological or Genetic Blockade of Endogenous De Novo Fatty Acid Synthesis Does Not Increase the Uptake of Exogenous Lipids in Ovarian Cancer Cells
Source: Front Oncol. 2021 Apr 13;11:610885. doi: 10.3389/fonc.2021.610885 (PMC8076863; doi:10.3389/fonc.2021.610885)
Supplement: Supplementary file 1 [file DataSheet_1.pdf]

SUPPLEMENTARY INFORMATION

The pharmacological or genetic blockade of endogenous *de novo* fatty acid synthesis does not increase the uptake of exogenous lipids in ovarian cancer cells

Thomas W. Grunt[1,2,3*], Lisa Lemberger[1], Ramón Colomer[4], María Luz López-Rodríguez[5], Renate Wagner[1,2]

[1]Cell Signaling and Metabolism Networks Program, Division of Oncology, Department of Medicine I, Medical University of Vienna, Waehringer Guertel 18-20, 1090 Vienna, Austria.

[2]Comprehensive Cancer Center, Vienna, Austria.

[3]Ludwig Boltzmann Institute for Hematology and Oncology, Vienna, Austria.

[4]Department of Medical Oncology, Hospital Universitario La Princesa and Spanish National Cancer Research Centre (CNIO), Clinical Research Program, Madrid, Spain.

[5]Departamento de Química Orgánica I, Facultad de Ciencias Químicas, Universidad Complutense de Madrid, Madrid, Spain.

*Correspondence:
Thomas W. Grunt, M.Sc., Ph.D.
Division of Oncology, Department of Medicine I
Medical University of Vienna
Waehringer Guertel 18 - 20
A-1090 Vienna, Austria
E: thomas.grunt@meduniwien.ac.at

Word count Abstract: 307

Word count main part (Introduction – Discussion): 4,236

Number of tables: 1

Number of figures: 6

Number of supplementary tables: 1

Number of supplementary figures: 4

Keywords: CD36, FASN inhibitors, fatty acid synthase (FASN), fatty acid uptake, LDL, lipid uptake, ovarian cancer, resistance

Running title: FASN-inhibition and lipid-uptake in ovarian-cancer

Supplementary materials and methods

Positive controls for Western blotting of FABP proteins

Absence of expression of FABP1, FABP2, FABP3, FABP7, FABP8, and FABP9 proteins in OC cell lines was confirmed by Western blotting using HEK293T cells transfected with plasmids expressing the respective FABP cDNAs (OriGene Technologies, Rockville, MD). Protein lysates were reconstituted in SDS sample buffer and subjected to SDS–PAGE along with cell lysates of untreated A2780, OVCAR3 and SKOV3 OC cell lines. Gels were blotted and co-immunostained using combinations of anti-actin with anti-FABP1, anti-FABP2, anti-FABP3, anti-FABP7, anti-FABP8, or anti-FABP9 before detection with peroxidase-labelled secondary antibodies and enhanced chemiluminescence reagents.

Cell cycle analysis

A2780 cells were grown for 48 hours in media containing 5% FCS or 5% lipid-depleted FCS, permeabilized in ethanol, stained with propidium iodide and analyzed for DNA content with a FACSCalibur flow cytometer (Becton-Dickinson, Franklin Lakes, NJ). Cell cycle distribution was calculated using ModFit LT™ software (Verity Software House, Topsham, ME) and cell counts were expressed in percent of total. Means ± SD of 3 independent experiments are given.

Apoptosis assay

Cells were cultured as described for cell cycle analysis, fixed in 2% formaldehyde and permeabilized in methanol. One million cells were stained in 100 µl of a proprietary solution containing pre-diluted phycoerythrin-labeled anti-active caspase 3 (Becton-Dickinson), and were analyzed in a FACSCalibur flow cytometer to determine the proportion of apoptotic cells that express active caspase 3 – a cleavage product of procaspase-3. FlowJo software (Becton-Dickinson) was used to determine the percentage of apoptotic cells in lipid-deprived cells compared to controls grown in the presence of lipids.

Supplementary figure legends

Supplementary figure S1

Full images of Western blot autoradiographs demonstrating partial knock-down of FASN protein expression 72 hours post-transfection of A2780 OC cells with FASN siRNAs (30nM) (left autoradiograph). Actin was used as loading control (right autoradiograph). One of three independent experiments is shown.

Supplementary figure S2

FASN inhibitors are potent growth inhibitors with low non-specific toxicity as determined by direct cell counting (A, B), viability dye exclusion (C, D), and formazan dye assay (E). A. OC cell lines A2780, OVCAR3 and SKOV3 were exposed for 24 hours to < 0.2% DMSO solvent (-FASN inhibitor), whereas experimental cultures (+FASN inhibitor) were treated with moderate concentrations of G28UCM (A2780: 10µM, OVCAR3: 30 µM, SKOV3: 20 µM) or Fasnall (A2780: 90 µM) before cells were trypsinized and counted in a hemocytometer. B. SKOV3 cells were exposed for the indicated periods of time to solvent or to a very high concentration (80 µM) of G28UCM before trypsinization and counting. Results are given in % of solvent control. C, D. Cells treated as described in A and B, respectively, were examined by trypan blue dye exclusion for determination of viability rates. Results are given in % viable cells. E. A2780 cells were exposed for 72 hours to solvent or to 1.25 µM TVB-3664 and cell growth was determined by formazan dye assay as described in the Materials and methods section. Results are given in % of solvent control. Means ± SD, n = 3. One-tailed Student's t-test (A, E) or ANOVA followed by Scheffe test (B, D). $p < 0.05$ (*), $p < 0.01$ (**), $p < 0.001$ (***) relative to controls.

Supplemental figure S3

Expression of lipid metabolic proteins in OC cell lines as determined by Western blotting and presented in form of full images of autoradiographs. A. Baseline levels of the lipogenic enzymes ACLY, ACC1, ACC2 and FASN, the lipid transport proteins FABP1 – FABP9, and the lipid receptor proteins CD36 and LDLR in A2780, OVCAR3 and SKOV3 OC cell lines. A2780 cells were found to express the widest range of these lipid metabolic proteins. Western blots of positive controls for those FABPs (FABP1, FABP2, FABP3, FABP7, FABP8, FABP9) that are not expressed in the OC cells are shown in the lower right corner. B, C. Inhibition of FA synthesis by administration of low (B) or high doses (C) of FASN inhibitors G28UCM or Fasnall does not affect the expression of the lipid metabolic proteins in A2780 OC cells. Blots were (co-)probed for several proteins of interest, stripped if necessary and re-probed. Actin was used as loading control. Three gels were used to cover all proteins of interest. Images of whole autoradiographs of all proteins of interest are shown in A, while images are shown in B and C only for those proteins that are expressed in the cells.

Supplemental figure S4

Cultivation of A2780 OC cells in lipid-free media down-regulates expression of lipid metabolic proteins as shown in full images of Western blot autoradiographs. Membranes were (co-)probed for several proteins of interest, stripped if necessary and re-probed. Actin was used as loading control. Three gels were used to cover all proteins of interest. Images of whole autoradiographs are shown only for those proteins that are expressed in the cells.
