# Supplementary figures and images for: The Pharmacological or Genetic Blockade of Endogenous De Novo Fatty Acid Synthesis Does Not Increase the Uptake of Exogenous Lipids in Ovarian Cancer Cells
Source: Front Oncol. 2021 Apr 13;11:610885. doi: 10.3389/fonc.2021.610885 (PMC8076863; doi:10.3389/fonc.2021.610885)

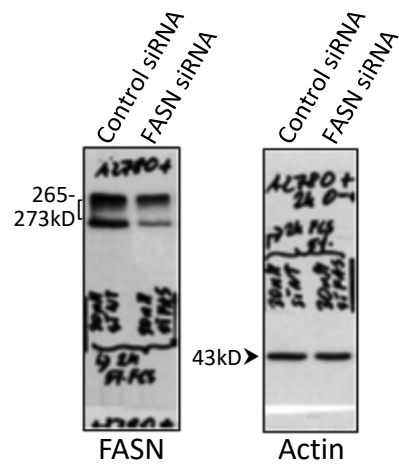

Supplement: Supplementary file 2 [file DataSheet_2.zip › Supplementary Figure 1.pdf]

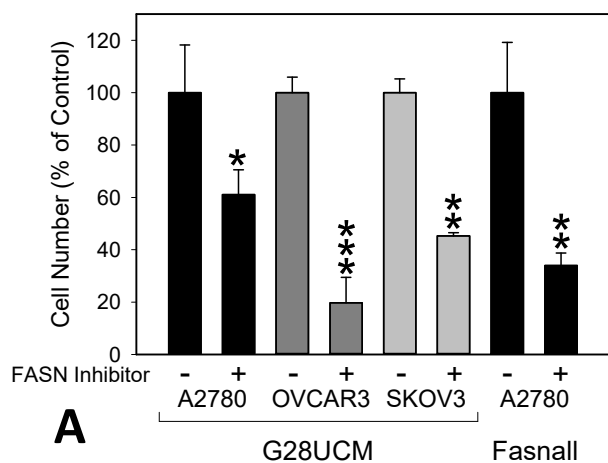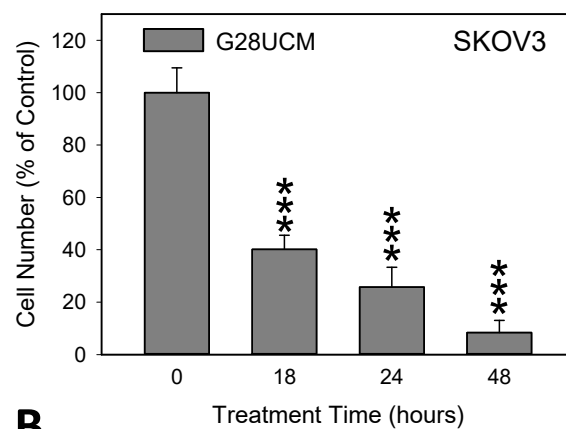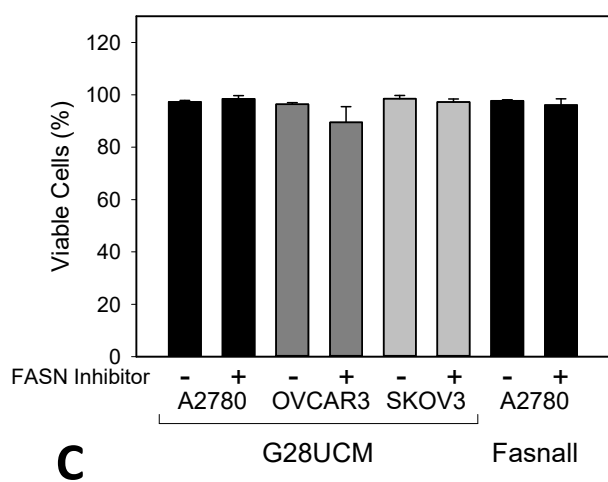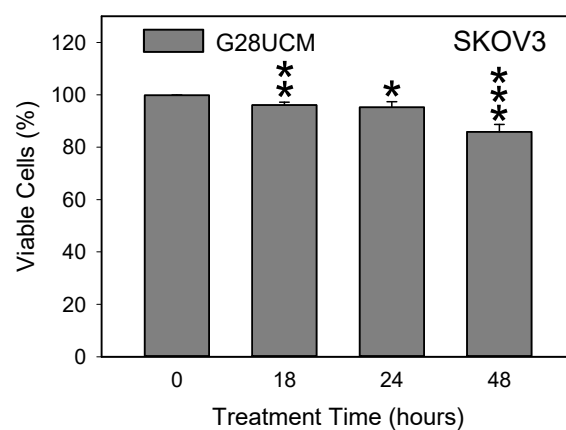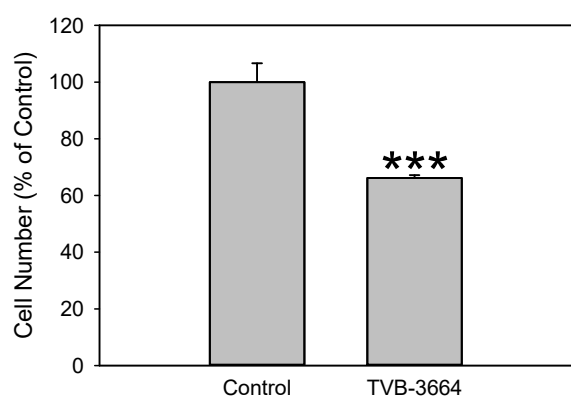

Supplementary Figure S2

Supplement: Supplementary file 2 [file DataSheet_2.zip › Supplementary Figure 2.pdf]

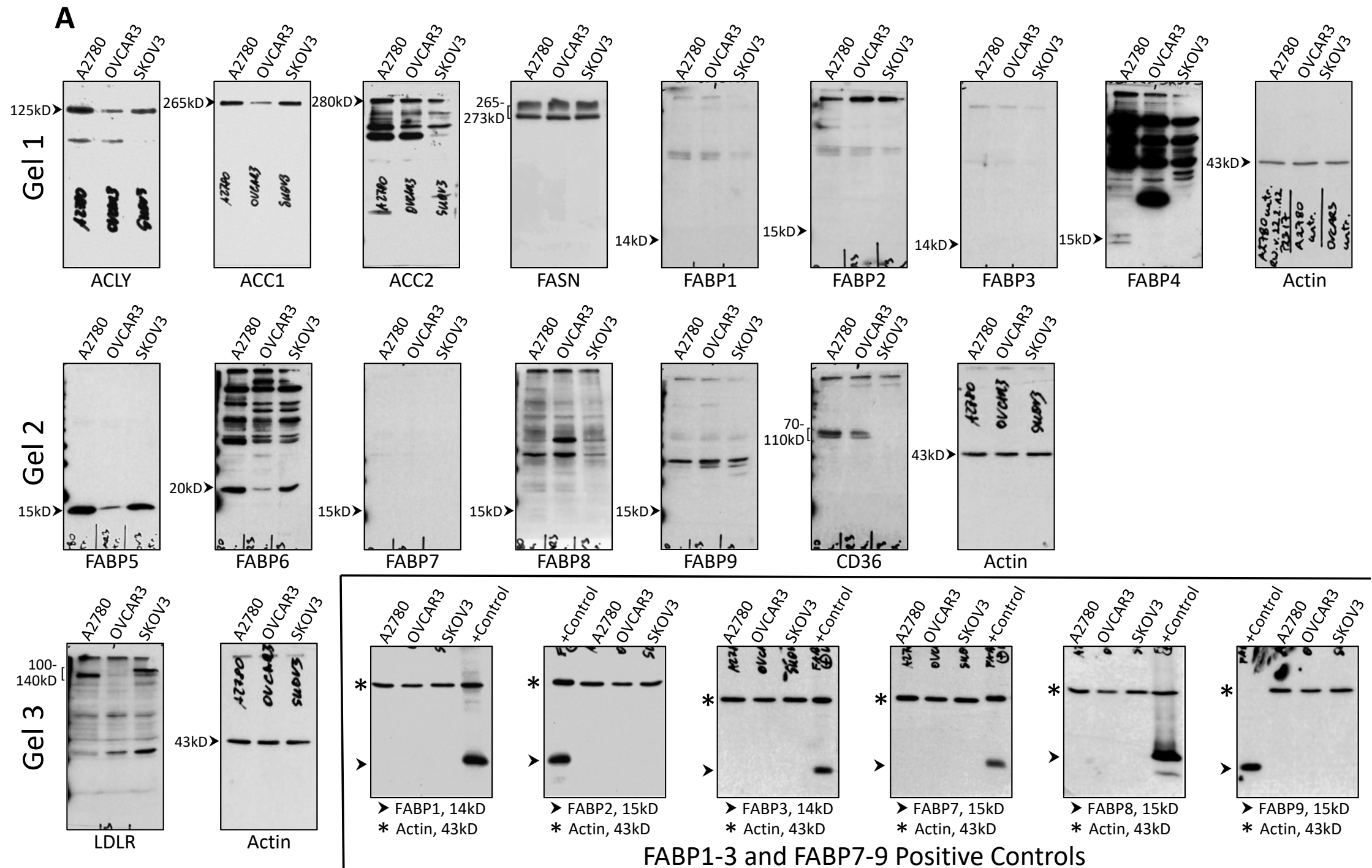

Supplementary Figure S3

Supplement: Supplementary file 2 [file DataSheet_2.zip › Supplementary Figure 3A.pdf]

**B**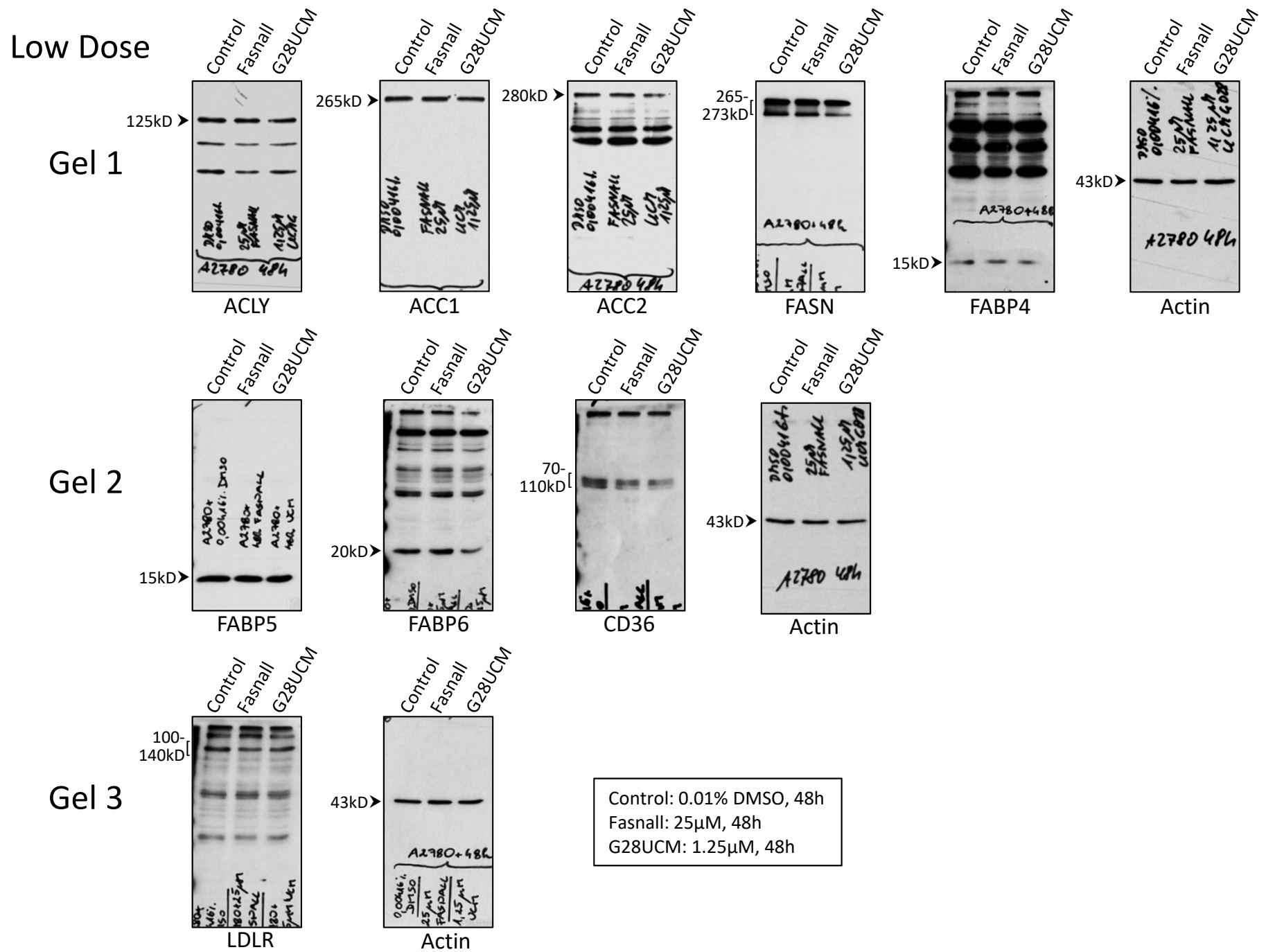

Supplementary Figure S3

Supplement: Supplementary file 2 [file DataSheet_2.zip › Supplementary Figure 3B.pdf]

C

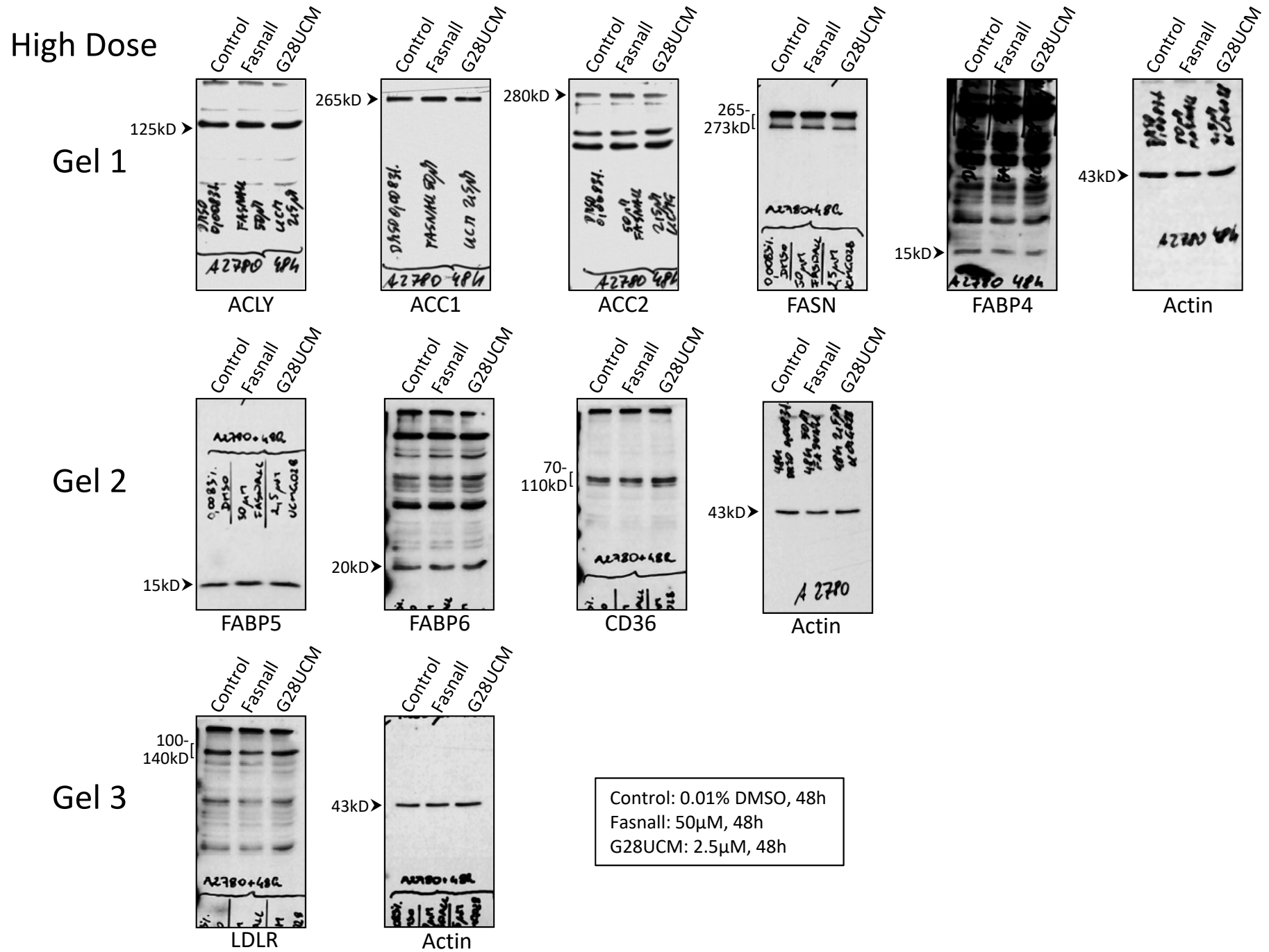

Supplementary Figure S3

Supplement: Supplementary file 2 [file DataSheet_2.zip › Supplementary Figure 3C.pdf]

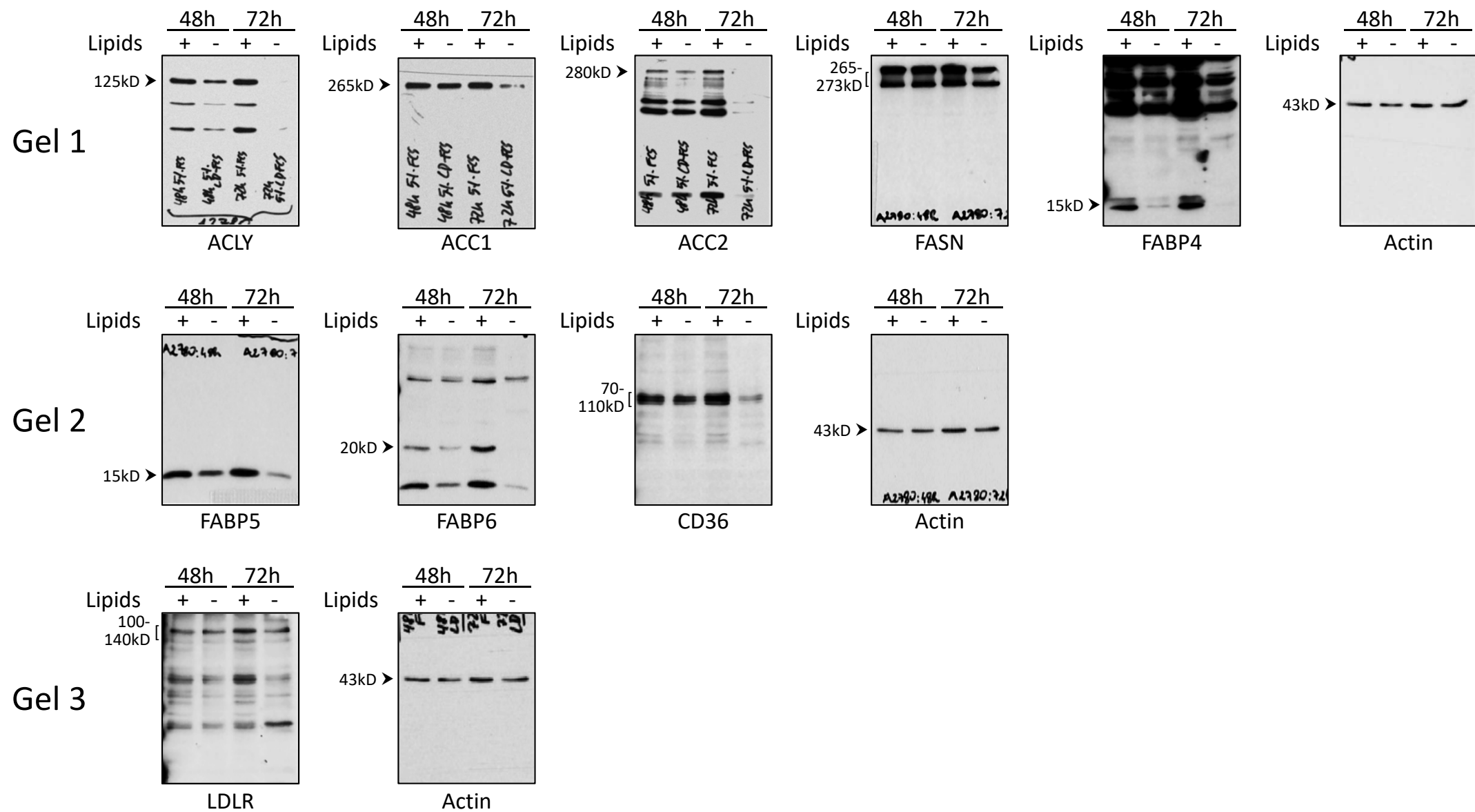

Supplementary Figure S4

Supplement: Supplementary file 2 [file DataSheet_2.zip › Supplementary Figure 4.pdf]
