# Supplementary material for: The Pharmacological or Genetic Blockade of Endogenous De Novo Fatty Acid Synthesis Does Not Increase the Uptake of Exogenous Lipids in Ovarian Cancer Cells
Source: Front Oncol. 2021 Apr 13;11:610885. doi: 10.3389/fonc.2021.610885 (PMC8076863; doi:10.3389/fonc.2021.610885)
Supplement: Supplementary file 3 [file Table_1.pdf]

Supplementary Table S1. Withdrawal of lipids from the serum of the culture medium causes neither cell cycle arrest nor apoptosis. Means ± SD of at least three independent experiments (for details see Supplementary materials and methods section).

	**Cell Cycle**			**Apoptosis**
	**G0/1 (%)**	**S (%)**	**G2/M (%)**	**Cleaved Caspase -3 Positive (%)**
**FCS + Lipids**	71,07 ± 3,85	21,69 ± 4,41	7,24 ± 1,06	1,65 ± 1,01
**FCS – Lipids**	71,85 ± 5,84	20,13 ± 2,77	8,02 ± 2,27	5,34 ± 2,40
